# Hepatocyte nuclear factor 4-α is necessary for high fat diet-induced pancreatic β-cell mass expansion and metabolic compensations

**DOI:** 10.3389/fendo.2024.1511813

**Published:** 2024-12-17

**Authors:** Francieli Caroline de Ramos, Robson Barth, Marcos Rizzon Santos, Milena dos Santos Almeida, Sandra Mara Ferreira, Alex Rafacho, Antônio Carlos Boschero, Gustavo Jorge dos Santos

**Affiliations:** ^1^ Islet Biology and Metabolism Lab – IBM Lab, Department of Physiological Sciences, Center of Biological Sciences, Federal University of Santa Catarina - UFSC, Florianópolis, Santa Catarina, Brazil; ^2^ Laboratory of Investigation in Chronic Diseases - LIDoC, Department of Physiological Sciences, Center of Biological Sciences, Federal University of Santa Catarina - UFSC, Florianópolis, Santa Catarina, Brazil; ^3^ Laboratory of Endocrine Pancreas And Metabolism - LAPEM, Department of Structural and Functional Biology, Institute of Biology, State University of Campinas - UNICAMP, Campinas, Brazil

**Keywords:** β-cell replacement, HNF4α, β-cell mass expansion, obesity, β-cell regeneration

## Abstract

**Aims:**

This study investigates the role of Hepatocyte Nuclear Factor 4α (HNF4α) in the adaptation of pancreatic β-cells to an HFD-induced obesogenic environment, focusing on β cell mass expansion and metabolic adaptations.

**Main methods:**

We utilized an HNF4α knockout (KO) mouse model, with CRE-recombinase enzyme activation confirmed through tamoxifen administration. KO and Control (CTL) mice were fed an HFD for 20 weeks. We monitored body weight, food intake, glucose tolerance, insulin sensitivity, and insulinemia. Also, to assess structural and metabolic changes, histological analyses of pancreatic islets and liver tissue were conducted.

**Key findings:**

KO mice displayed lower fasting blood glucose levels compared to CTL mice after tamoxifen administration, indicating impaired glucose-regulated insulin secretion. HFD-fed KO mice consumed less food but exhibited greater weight gain and perigonadal fat accumulation, reflecting higher energy efficiency. Histological analysis revealed more pronounced liver steatosis and fibrosis in KO mice on HFD. Glucose intolerance and insulin resistance were exacerbated in KO mice, highlighting their inability to adapt to increased metabolic demand. Structural analysis showed that KO mice failed to exhibit HFD-induced β cell mass expansion, resulting in reduced islet diameter and number, confirming the critical role of HNF4α in β cell adaptation.

**Significance:**

This study demonstrates that HNF4α is essential for the proper metabolic and structural adaptation of pancreatic β-cells in response to an obesogenic environment. The lack of HNF4α impairs β cell functionality, leading to increased susceptibility to glucose intolerance and insulin resistance. These findings underscore the importance of HNF4α in maintaining glucose homeostasis and highlight its potential as a therapeutic target for diabetes management in obesity.

## Introduction

Diabetes Mellitus (DM) is a metabolic disorder characterized by chronic hyperglycemia and presents multiple etiologies; this disease affects more than 350 million people and is considered a major public health concern and a risk factor for other complications, such as retinopathy and kidney injury (1). This illness may arise from absolute insulin deficiency due to a combined effect of a genetic predisposition with an autoimmune-induced near-to-total β cell destruction (Type 1 DM). It may also emerge from insulin resistance (IR) and reduced β cell function, which are usually related to obesity and sedentary lifestyle (Type 2 DM) (2, 3). Additionally, to these polygenic types of DM, there is a form of DM known as Maturity Onset Diabetes of the Young (MODY), which is monogenic and involves a single genetic mutation leading to defective glucose-induced insulin secretion (4). MODY is often linked to mutations in at least 16 different genes, including the Hepatocyte Nuclear Factor 4α (HNF4α) gene responsible for MODY1, characterized by asymptomatic hyperglycemia in children, adolescents or young adults (5).

HNF4α is a transcription factor (TF), a member of a nuclear receptor family, which possesses a zinc finger domain that binds DNA as a homodimer and plays a role in the development of the liver, kidneys, and intestines. In pancreatic β-cells, the significance of HNF4α is highlighted in the development of MODY1 (6), a type of DM resulting from a mutation in this transcription factor, which is responsible for various functions in β-cells, such as glucose metabolism and insulin production/secretion (7). HNF4α also regulates differentiation, proliferation, and growth processes in pancreatic β-cells, especially during the neonatal phase and pregnancy (8). Similarly, we have previously demonstrated that HNF4α is essential for dexamethasone-induced β cell mass expansion (9), highlighting HNF4α’s critical role in pancreatic adaptation to metabolic challenges.

Obesity is a significant risk factor for the development of DM2 and it is characterized by an excessive accumulation of fat due to an imbalance between energy intake and expenditure and is influenced by genetic, epigenetic, physiological, behavioral, and environmental factors. The interplay between obesity and IR involves complex metabolic and inflammatory processes. Obesity is associated with chronic inflammation, characterized by the infiltration of immune cells that release pro-inflammatory cytokines such as Tumor Necrosis Factor-α (TNF-α) and Interleukin-6 (IL-6) (10, 11). These cytokines can interfere with insulin signaling, contributing to insulin resistance (IR) (12). Additionally, a high-fat diet exacerbates this inflammatory state by promoting the growth of gram-negative bacteria in the gut, which produce inflammatory molecules such as Lipopolysaccharide (LPS) (13) leading to an IR state (14).

Due to its critical role in metabolism, especially in maintaining glycemic homeostasis and its relationship with the development of diabetes mellitus (DM), the pancreatic islet is extensively studied, with a particular focus on the β-cells, which are predominantly affected in DM pathogenesis. It is well established that pancreatic β-cells exhibit cellular plasticity, allowing them to adapt through differentiation, proliferation, and transdifferentiation processes to various conditions such as obesity, DM, and pregnancy. This cellular plasticity is regulated by several transcription factors, including HNF4α, with IR being a significant trigger for this process (15). Regardless of its etiology, all types of DM involve a reduction in insulin-secreting cell mass. Therefore, it is important to investigate pathways that increase this cell mass to identify potential molecular targets for DM treatment.

Here, we have shown for the first time that HNF4α is crucial for the β-cell mass expansion induced by High-fat Diet (HFD) pointing this TF out as a target for the DM treatment.

## Materials and methods

### Ethics statement

The experimental protocol was approved by the Federal University of Santa Catarina Committee for Ethics in Animal Experimentation (identification no 7696221019) in accordance with the Brazilian National Council for Animal Experimentation Control (CONCEA) and the Guide for the Care and Use of Laboratory Animals (National Research Council, 9th edition).

### Animals

All mice were maintained in appropriate cages at the Physiological Science Department in a temperature (22–24 °C) and humidity-controlled environment and kept on a 12-h light-dark cycle (lights on 06:00–18:00 h) with access to food (Nuvilab CR1, Brazil) and water (filtered tap) ad libitum. To test our hypothesis we used tissue-specific and temporal-conditional knockout male mice for HNF4α (HNF4αloxP/loxP;Ins1Cre+), generated by crossing the lineage carrying the CRE-recombinase enzyme in β-cells (Ins2.Cre+- #008122, Jackson Laboratory, USA) and the lineage that possesses the gene for the HNF4α FT flanked by the LoxP sequence (HNF4αloxP/loxP - #004665, Jackson Laboratory, USA). The KO mice were homozygous for the loxP-flanked allele and heterozygous for the RIP-CreER, while the control (CTL) mice were homozygous for the loxP-flanked allele but did not carry the RIP-CreER. To determine the genotype of the mice, RT-PCR was performed according to the Jackson Laboratory instructions. To activate the CRE-recombinase enzyme, mice aged 35-40 days received daily intraperitoneal injections of 100 µl of a 20 mg/mL tamoxifen solution (#T5648, Sigma-Aldrich, USA) for five consecutive days (9).

### Experimental design

To confirm the CRE-recombinase activation, we evaluated the fasted glycaemia 15 days after the last tamoxifen injection. After that, mice were divided into three groups: Control (CTL) fed with a chow diet, Control fed with HFD, and Knockout fed with HFD. The HFD administration lasted for 20 weeks, and the compositions of the diets are shown in **Table 1**.

**Table 1:** Diet composition.

Ingredients	Standard Diet *(*g/Kg)	High-fat Diet *(*g/Kg)
Casein	140	200
Starch	115.5	115.5
Dextrain	155	132
Sucrose	100	100
L-cystin	1,8	3
Fiber	50	50
Soy Oil	40	40
Lard	0	312
Salts AIN93G (17)	35	35
Vitaminas AIN93G (17)	10	10
Choline hydrochloride	2.5	2.5
Energy content	3.69 kcal/g	5.29 kcal/g

### Pancreatic islet isolation and qPCR

After euthanasia, the pancreas was injected with Hank’s buffer containing 0.9 mg/ml of type V collagenase. Once removed, the inflated pancreas was incubated for 17 minutes at 37°C and the islet was hand-picked one-by-one. To analyze the HNF4α mRNA, 200 islet were homogenized with Tryzol™ (Invitrogen, MA, USA) and the mRNA was isolated following the manufacture’s guide. After the extraction, 200 ng of mRNA were converted into cDNA with the HighCapacity™ cDNA Transcription Kit (Applied Biosystems, CA, USA). The qPCR was performed with the Fast SYBR™ Green Master Mix (AppliedBiosystem, MA, USA) and the GAPDH gene was used as endogenous control. The primers used are GAPDH Forward: CACATTGGGGGTAGGAACAC; GAPDH Reverse: GCCAAAAGGGTCATCATCTC; HNF4α Forward: GCAAGTGAGCCTGGAGGATT; HNF4α Reverse: TGTCCATTGCTGAGGTGAGA.

### Intraperitoneal glucose tolerance tests

Ten-hours fasted mice received an intraperitoneal administration of 1 g/kg of glucose dissolved in saline solution (0.9% NaCl wt/vol). Blood glucose levels were measured before (0 min) and 15, 30, 60, and 120 minutes after glucose administration. Glucose levels were evaluated in blood drops collected from the tail tip and measured by Accu-Chek Performa II glucometer (Roche^®^).

### Intraperitoneal insulin tolerance tests and glucose decay constant

One-hour fasted mice received an intraperitoneal administration of 1 IU/kg insulin dissolved in saline solution (0.9% NaCl wt/vol). Blood glucose levels were measured before (0 min) and 5, 15, 30, 45 and 60 minutes after the insulin administration. Glucose levels were evaluated in blood drops collected from the tail tip and measured by Accu-Chek Performa II glucometer (Roche^®^). The rate of glucose decay constant (kITT) was calculated from the blood glucose concentrations during the linear decay phase, using the formula 0.693/t1/2.

### Fasting insulinemia

Before the euthanasia, the mice were 10-hours fasted and the trunk blood samples were collected and placed into microtubes containing anticoagulant heparin. The tubes were centrifuged at 1100 G, 15 min, 4°C, and the plasma was collected and stored at −80°C. Insulin was measured by kit AlphaLISA Detection Kit. (#AL204C, PerkinElmer^®^, MA, USA), as described according to the manufacturer’s instructions.

### Immunofluorescence of pancreatic tissue

After euthanasia the pancreas were dissected, weighed and fixed in paraformaldehyde 4% w/v for 24 hours at room temperature. Then, the organ was dehydrated and embedded in paraffin. A serial section of five µm-thickness were done and placed into a silanized slide. After deparaffinization, the sections were rehydrated and incubated in 10mM sodium citrate solution (pH 6) at 98°C for 30 min. After the antigenic retrieval, permeabilization and the blocking of unspecific bindings, the slides were incubated with primary antibody anti-insulin A0564, Dako cytomation, CA, USA, and anti-glucagon sc7779R, Santa Cruz Biotechnology (at dilution 1:100 and 1:25 respectively, in PBS 1% bovine albumin) for 4 hours at 4°C in humidity chamber. After this period, the sections were washed in PBS and incubated with secondary anti-goat antibody AlexaFluor 488 (1:500) for 1 hour at room temperature. Then, the sections were washed 3 more times in PBS, incubated with DAPI for five minutes in a moisture chamber and then mounted with coverslips using Vectashield antifading mounting media. The images were obtained by AxioScan (ZEISS^®^, Oberkochen, Germany). The islet morphology analysis was done as described before (16). Representative images were obtained using an inverted microscope (OLYMPUS^®^; Tokyo, Japan).

### Liver processing and hematoxylin-eosin staining

After euthanasia, the liver was extracted and fixed in paraformaldehyde 4% w/v for 24 hours at room temperature. After this period, the organ was dehydrated and embedded in paraffin. Five µm-thickness sections were done and placed in silanized slide. After the deparaffinization, the five µm-thickness sections were hydrated and stained with Ehrlich’s hematoxylin and eosin, acid and basic differentiator, dehydration and mounted with Entellan. Images were obtained using an inverted microscope (OLYMPUS^®^; Tokyo, Japan).

### Perigonadal fat processing

After euthanasia both perigonadal fat pad, right and left, was removed, weighed and the values was recorded.

### Statistical analysis

Groups were compared by one-way ANOVA using the unpaired Turkey’s *post-hoc* or Kruskal Wallis Test, with Dunn comparisons, when data were not parametric. Data are presented as the mean ± S.E.M. All data were considered statistically different if P value was ≤0.05.

## Results

### HNF4α knockout model validation

**Figure 1:**
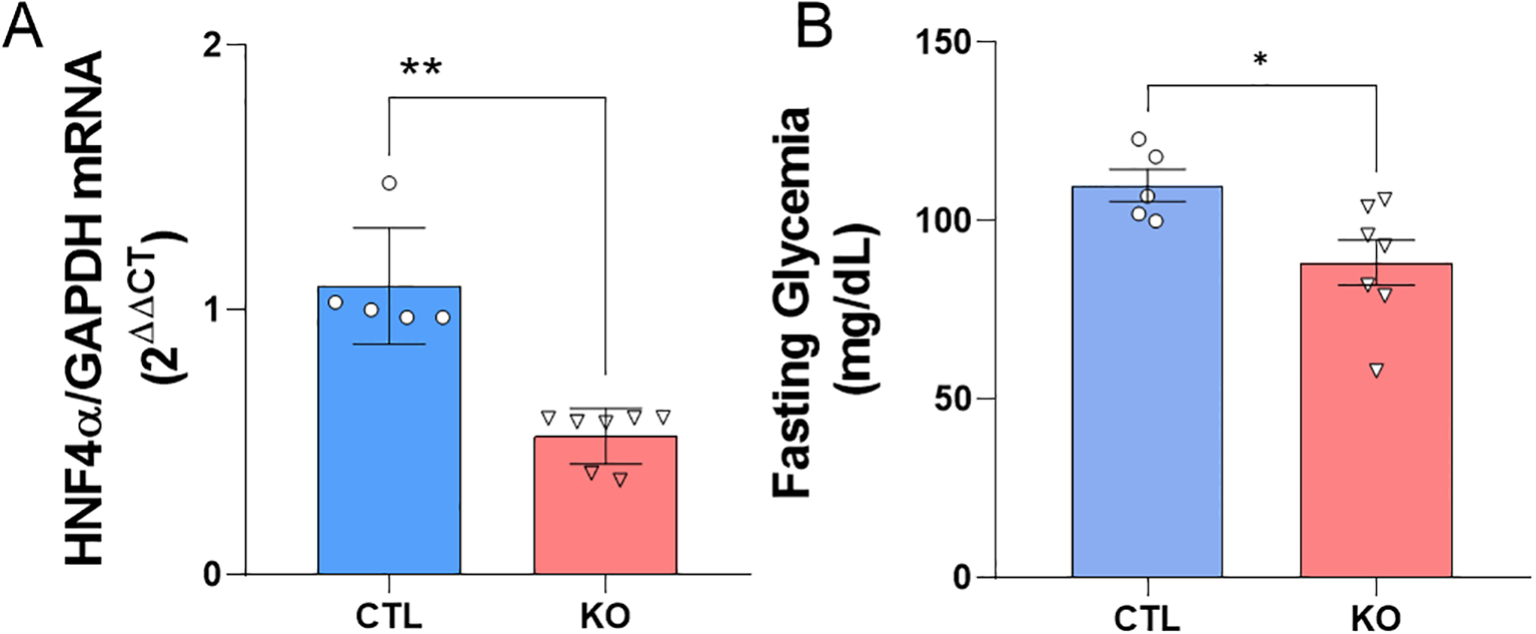
HNF4α knockout validation. **(A)** HNF4α mRNA expression. The data are presented as HNF4α/GAPDH gene expression and related to CTL. **(B)** Fasting blood glucose of Knockout and Control mice before exposure to the high-fat diet or control diet. Blood glucose analysis after 10 hours of fasting of CTL mice: n=5 and KO n=7, 15 days after the last application of tamoxifen. CTL: HNF4αloxP/loxP;Ins1Cre- and KO: HNF4αloxP/loxP;Ins1Cre+. Data is shown as mean ± SEM. P-value presented as **p<0.005 and *p<0.05 comparisons using Student’s t-test.

To validate the HNF4α knockout model we analyzed the mRNA for the transcription factor HNF4α in isolated islet from CTL and KO mice. As expected, we found a reduction near to 50% in the HNF4α mRNA expression in KO mice (**Figure 1A**). To confirm the CRE-recombinase activation, we assessed the fasting glycaemia and, it was lower in KO compared to CTL mice (**Figure 1B**).

### HFD outcomes

**Figure 2:**
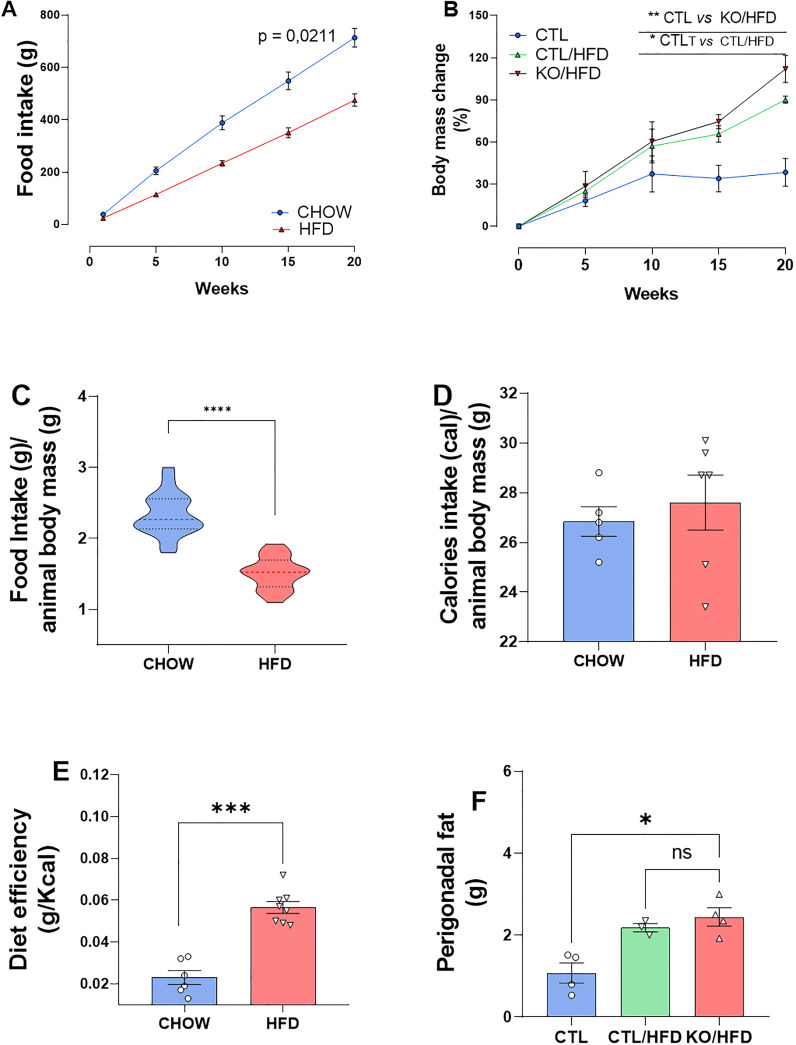
Cumulative diet intake, caloric intake, caloric efficiency and food intake relative to the animals’ body mass, body mass gain and adipose tissue in response to exposure to a high-fat diet in knockout and CTL animals. Standard diet intake: n = 4 and HFD: n = 4 accumulated throughout the experiment **(A)**. Standard diet intake: n = 4 and HFD: n = 4 normalized with animal mass **(B)**. Daily calories consumed per animal standard diet: n = 6 HFD: n=8 **(C)**. Standard diet efficiency: 6 HFD: 8 obtained through the ratio between the animal’s Δ mass (g) and caloric intake (Kcal) **(D)**. Percentage of CTL body mass change: n = 3; CTL/HFD: n=3; KO/HFD: n=4 **(E)**. Accumulation of perigonadal adipose tissue in response to exposure to high-fat diet or standard diet: CTL: n=3; CTL/HFD n=3; KO/HFD = 4 **(F)**. Data shown as mean ± SEM. p values presented as *p<0.05, **p<0.005, ***p<0.001, ****p<0.0001, using Mann Whitney test for food efficiency of the diet and accumulated food intake, Student’s t-test for intake accumulated food and normalized by the mass of the animal. Using Kruskal Wallis Test with Dunn comparisons for perigonadal fat.

**Figure 3:**
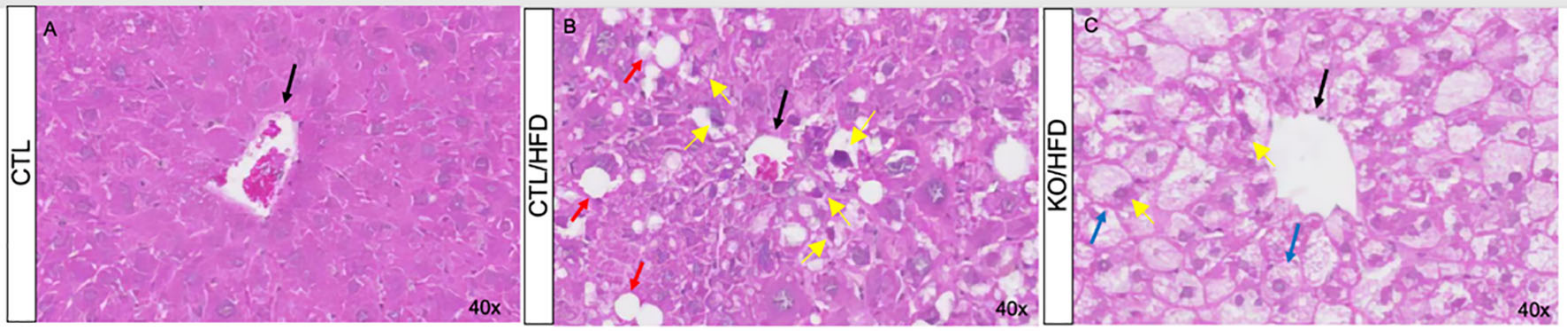
The effect of the high-fat diet and control diet observed in histological sections of liver tissue from CTL and Knockout mice for HNF4α. Hematoxylin-Eosin staining of liver tissue from CTL **(A, B)** and Knockout **(C)** animals. Animals fed standard diet for 20 weeks **(A)** or HFD **(B, C)**. The black arrows indicate the central lobular veins, red arrows highlight accumulation of fat (steatosis), blue arrows indicate bulging hepatocytes due to fat storage and, yellow arrows indicate Mallory hyaline.

During the 20-week HFD regiment, HFD-fed mice consumed less amount of food than CHOW-fed mice (**Figures 2A, C**). Daily caloric intake was similar across groups (**Figure 2D**), but HFD-fed mice showed greater energy efficiency, leading to increased body mass and perigonadal fat (**Figures 2E, B, F**). Liver analysis showed higher lipid accumulation in KO/HFD mice, with larger lipid droplets, Mallory hyaline, and hepatocyte bulging, suggesting more pronounced steatosis and fibrosis compared to CTL mice (**Figure 3**).

### Metabolic adaptation in obesity

**Figure 4:**
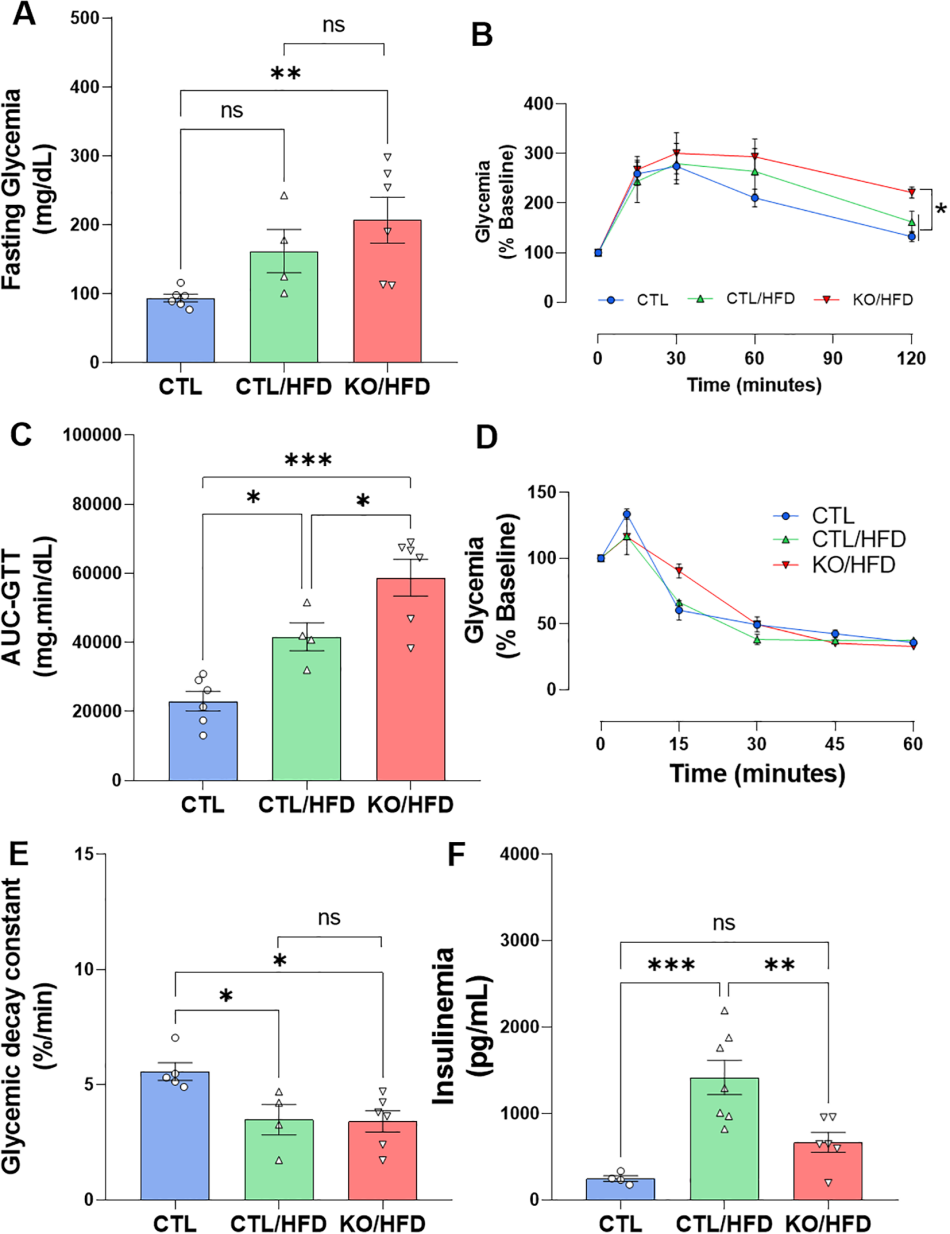
HNF4α KO exhibit reduced metabolic adaptation when exposed to HFD. Fasting blood glucose before ipGTT **(A)** and analysis of glucose tolerance test with intraperitoneal application of 1 U/kg dextrose, ipGTT of CTL mice: n = 6; CTL/HFD n = 4 KO/HFD: n = 6 after 20 weeks of dietary exposure **(B)** The area under curve (AUC) was derived from the ipGTT plot **(C)**. Insulin tolerance test, application of 1 U/kg intraperitoneally, collection of caudal blood, ipITT. CTL: n = 5; CTL/HFD: n = 4; KO/HFD n = 6 **(D)**. Glucose decay constant in response to application, kITT, graph derived from ITT data, CTL: n = 5; CTL/HFD: n = 4; KO/HFD n = 6 **(E)**. Insulinemia after 10h of fasting, stem blood CTL: n = 4, CTL/HFD: n = 7, KO/HFD: n = 6 **(F)** Data shown with mean ± SEM **(A, D–F)**. p-values presented as *p<0.05, **p < 0.01, ***p < 0.001 using 1-way ANOVA with Tukey *post hoc* for fasting blood glucose, AUC, blood glucose decay constant, ipGTT and using Kruskal Wallis test with Dunn comparisons for fasting insulinemia.

KO mice fed with HFD had significantly higher fasting blood glucose levels than CTL mice and was not different from CTL/HFD, this may represent an additive effect of diet and genotype (**Figure 4A**). Also, KO/HFD mice presented greater glucose intolerance compared with CTL and CTL/HFD mice (**Figures 4B, C**). Insulin sensitivity was reduced in both CTL/HFD and KO/HFD group (**Figure 4E**), but fasting insulinemia was lower in KO/HFD mice compared to CTL/HFD mice (**Figure 4F**).

### Structural islet adaptation in obesity

**Figure 5:**
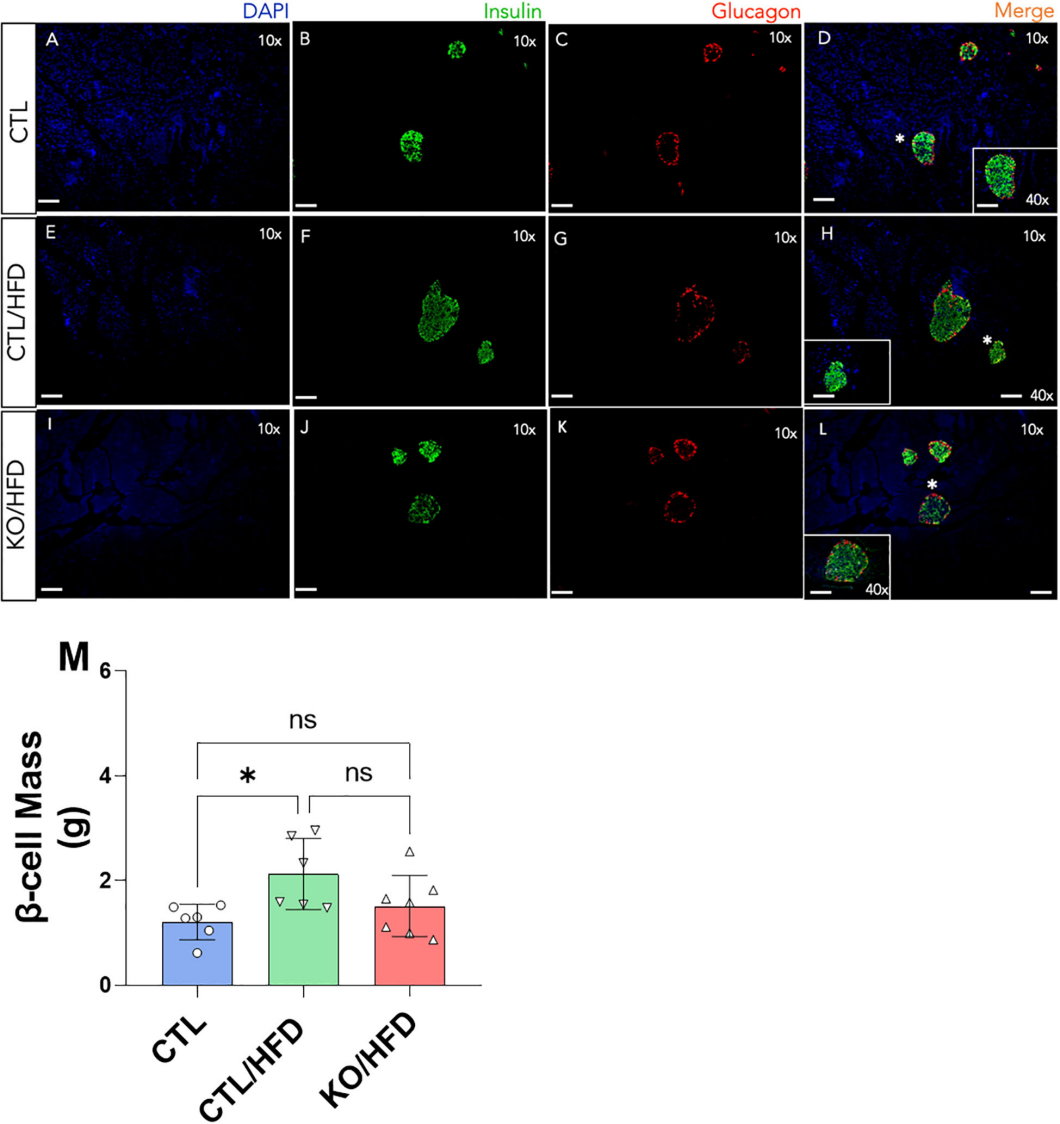
Immunofluorescence of the pancreas of Knockout and Control animals fed or not with an HFD diet and quantitative analysis of β cell mass from the Immunofluorescence data. Scale bars mean 100 µm at a final magnitude of 10x and 40x, as indicated in panels **(A-L)** Cell mass calculation β was made in duplicate from two different sections of the pancreas of the same animal CTL: n = 3, CTL/HFD: n = 3, KO/HFD: n = 5 **(M)**. Mean ± SEM. p values presented as *p<0.05, 1-way ANOVA with Tukey *post hoc*.

The HFD-induced increase in β-cell mass expansion was only observed in CTL/HFD mice (**Figure 5**). Additionally, islet diameter was significantly smaller in KO/HFD mice compared to CTL/HFD mice (**Figure 5**).

## Discussion

### HNF4α Knockout model validation

Since the number and/or the functionality of the pancreatic β-cells is the root of Diabetes mellitus, regardless of the etiological classification (DM1, DM2) of this disease, any kind of treatment that restores a functional β-cell mass will benefit diabetic patients. The Transcription Factor HNF4α is pointed out as a relevant target that could be manipulated (6, 9) to aim β-cell regeneration. So, in this scenario and based on the fact that HNF4α may participate in the metabolic-induced pancreas adaptation, we decided to investigate the HNF4α role on the pancreatic β-cells and metabolism adaptation to an HFD-induced obesogenic environment.

Before exposing the mice to an obesogenic environment, we validated the mice model that lack HNF4α expression specifically in pancreatic β-cell. As expected, we found a reduction near 50% in the HNF4α mRNA expression in KO mice compared to CTL mice (**Figure 1A**). It is worth to point that the expression of HNF4α is not limited to β-cells and our analysis was done in the whole islet.

Pancreatic β-cells are a great sensor of glycemia; therefore, small changes in physiological glucose concentration influence insulin secretion. This ability is due to the function of several enzymes and proteins, such as glucose transporter 2 (GLUT2), glucokinase (GCK), and ATP-sensitive potassium channel (KATP). Once glucose enters β-cells via GLUT2, it is phosphorylated by glucokinase (GCK) to form glucose-6-phosphate. This molecule then undergoes glycolysis to produce pyruvate, which enters the tricarboxylic acid (TCA) cycle, ultimately increasing ATP production (18). The resulting rise in the ATP/ADP ratio leads to the closure of KATP channels, whose functionality depends on the proper expression of the Kir6.2 subunit, regulated by the HNF4α gene. Closure of the KATP channels causes membrane depolarization which, in turn, opens voltage-dependent L-type Ca2+ channels, allowing a rapid influx of Ca2+ into the cell. The increase in intracellular cytosolic Ca2+ is the primary mediator of insulin exocytosis (19).

To confirm the activation of the CRE-recombinase enzyme, we evaluated the fasting blood glucose levels of the mice 15 days after the final tamoxifen administration. As expected (9), KO mice showed lower fasting blood glucose levels compared to CTL mice (**Figure 1B**). Since KATP channels are an important key in the β cell’s glucose sensing ability and that the Kir6.2 gene, a subunit of the KATP channel, is controlled by the HNF4α, the KO mice β-cells present an inability to secrete insulin in a regulated manner, resulting in lower insulin secretion under basal blood glucose conditions (fasting glycemia).

In addition, HNF4α also contributes to the expression of mRNAs for genes involved in glucose metabolism and glucose-stimulated insulin secretion (GSIS). We have previously shown that HNF4α KO mice present higher insulinemia before (0’) and 60 min after glucose injection in the ipGTT. In addition, these KO animals present and increased GSIS that explains the lower fasting glycemia observed here and it confirms our HNF4α KO model (9).

### HFD outcomes

Upon the HNF4α KO model confirmation, we feed the mice HFD for twenty weeks. During this period, the mice were weighed, and their diet consumption was documented, wherein we noticed that the HFD-fed group had lower dietary intake during the course of the experiment compared to the CHOW-fed group. (**Figures 2A, B**).

Gastrointestinal hormones stimulated by fatty meals, such as Cholecystokinin (CCK), contribute to energy homeostasis, and some of these hormones may influence the brain by serving as satiety signals to inhibit food intake. Peripheral CCK is secreted by intestinal I cells in response to fatty meals and is involved in intestinal modulation, motility, stimulating pancreatic enzyme secretion, and regulating appetite (20). Intraperitoneal administration of purified CCK in fasted rats reduced food intake amount but not meal frequency, classifying CCK as a satiety peptide (21). Thus, in line with the literature and considering that it is physiological that the rodents in the food ingestion based on the energetic density of the diet (22), the HFD induced greater satiety than CHOW, resulting in animals consuming less food over the 20-week period.

Furthermore, despite **Figure 2C** (**Figure 2C**) suggests that the daily calorie consumption of both groups was similar, **Figure 2D** shows that HFD had a more pronounced energy efficiency than CHOW, justifying the greater body mass gain and increased perigonadal fat in mice fed an HFD (**Figures 2E, F**). In addition, the HFD-fed mice developed obesity, since they gained more weight and the excess energy was stored as fat.

The high-fat diet (HFD) also affects liver tissue. Our observations indicate that while HFD induces an increase in lipid storage in the liver regardless of genotype (**Figure 3**), knockout mice fed with HFD showed a significantly higher amount of lipid droplets in the liver (**Figures 3B, C**), along with alterations in Mallory hyaline and bulging hepatocytes, indicating a state of steatosis (23, 24). Additionally, the HFD-induced increase in liver parenchymal fibrosis was greater in animals lacking HNF4α compared to CTL mice (**Figure 3**).

These HFD outcomes, observed here, confirm that we have an obesity mice model and lead us to believe that the specific deletion of TF HNF4α in the β-cells of the pancreas may trigger systemic changes that are important for an adaptive response to obesity, probably due to the lack of the expected physiological amount circulating insulin in the knockout mice.

### Metabolic adaptation in obesity

To deeply investigate the metabolic adaptive response to obesity we evaluated the glucose tolerance, insulin sensitivity, and insulinemia after 20 weeks on the HDF feeding program. The HFD-induced increase in fasting glycaemia was greater in the KO mice than in the CTL mice (**Figure 4A**). Similarly, the glucose intolerance observed in the HFD fed mice was more prominent in the mice which lack the HNF4α in the β-cells. These results are in accordance with the literature (6) and show that the β-cell ability to respond to a glucose challenge is diminished, suggesting that KO mice are more susceptible to the effects of HFD, exhibiting, in addition to a significant increase in fasting blood glucose, a profound glucose intolerance (**Figures 4B, C**).

As expected, exposure to an HFD reduced insulin sensitivity in these mice, regardless of the genotype (**Figures 4D, E**). It is well established that any reduction in peripheral insulin sensitivity is adaptively compensated by increased pancreatic β-cell function. As expected, CTL mice fed with HFD presented an increase in fasting insulinemia, and such an expected outcome was not observed with the same magnitude in KO/HFD mice (**Figure 4F**).

When β-cells cannot adjust to the increased insulin demand imposed by factors that increase metabolic demand, such as glucocorticoids (25), pregnancy (6), or obesity (26) fasting and/or fed hyperglycemia may occur. Here, we showed that the lack of the TF HNF4α, specifically in β-cells, impaired the proper metabolic adaptation in an obesogenic environment due to a failure in the β-cells ability in respond to this scenario.

### Structural islet adaptation in obesity

To investigate if the inability of the β cell lacking HNF4α in response to obesity was due to a structural adaptation failure we analyzed the islet architecture and observed that the HFD-induced increase in β-cell mass expansion was not noticed in KO/HFD mice. Moreover, it is visible that the islet diameter was lower in the KO/HFD when compared with CTL/HFD group (**Figure 5**). These results corroborate the ones published by Gupta (6) and our group (9) and reinforce the evidence that the TF HNF4α is relevant for the ability of the β-cells to adapt themselves to respond to a scenario where there is an increase in the metabolic demand.

## Conclusion

This study underscores the critical role of Hepatocyte Nuclear Factor 4α (HNF4α) in the adaptive response of pancreatic β-cells to HFD-induced metabolic stress. Our findings demonstrate, for the very first time, that HNF4α knockout (KO) mice exhibit significant impairments in glucose-regulated insulin secretion, β cell mass expansion, and overall metabolic adaptation when exposed to an obesogenic environment. The inability of KO mice to properly regulate fasting blood glucose and respond to increased metabolic demand highlights the indispensable role of HNF4α in maintaining glucose homeostasis. Furthermore, the pronounced liver steatosis and fibrosis observed in HFD-fed KO mice suggest that HNF4α is crucial not only for pancreatic function but also for broader metabolic regulation. These insights provide a deeper understanding of the molecular mechanisms underlying β cell dysfunction in obesity and diabetes, emphasizing HNF4α as a potential therapeutic target for improving β cell resilience and function in metabolic diseases.

## Data Availability

The raw data supporting the conclusions of this article will be made available by the authors, without undue reservation.
